# Diversity of Secondary Metabolites in Roots from *Conium maculatum* L.

**DOI:** 10.3390/plants9080939

**Published:** 2020-07-24

**Authors:** Remigius Chizzola, Ulrike Lohwasser

**Affiliations:** 1 Institute of Animal Nutrition and Functional Plant Compounds, University of Veterinary Medicine Vienna, Veterinaerplatz 1, 1210 Vienna, Austria; 2 Leibniz Institute of Plant Genetics and Crop Research (IPK), Corrensstraße 3, 06466 Seeland, OT Gatersleben, Germany; lohwasse@ipk-gatersleben.de

**Keywords:** *Conium maculatum*, tap root, furocoumarins, xanthotoxin, isopimpinellin, falcarinol, falcarindiol

## Abstract

Background: *Conium maculatum* is known as highly toxic plant, due to piperidine alkaloids present in the aerial parts. In a first attempt, in various tap root samples, however, alkaloids could not be detected. The present study describes active compounds in the tap roots from 16 populations harvested at maturity. The compounds were extracted with dichloromethane from root pieces of single plants and analyzed by gas chromatography–mass spectrometry. Ten bioactive compounds were evaluated: five furocoumarins, two prenylated coumarins, two aliphatic C_17_-polyacetylenes and the phenylpropanoid elemicin. A high variability could be observed, the highest concentrations were measured for falcarindiol, xanthotoxin and isopimpinellin, the lowest for elemicin. In sum *C. maculatum* roots contained comparable amounts of compounds that are characteristic for Apiaceae, and also occur in vegetables as carrots, parsnip, parsley or celeriac.

## 1. Introduction

*Conium maculatum* L., poison hemlock, (Apiaceae) is a poisonous plant well known since antiquity, with high toxicity for animals and humans [1,2,3]. The plant grows preferentially on uncultivated land on loamy and nitrogen rich soils and is sparsely distributed widely throughout Europe and the Mediterranean region. In the aerial parts piperidine alkaloids as coniine and γ-coniceine are the toxic compounds. A further novel alkaloid, named conmaculin, has been described by Radulovic et al. [4]. Alkaloid content showed geographical variation and was influenced by herbivory [5].

Aerial parts may also contain some volatiles with the β-ocimene isomers and germacrene D being the main compounds [6]. Masoudi et al. mentioned germacrene D, β-caryophyllene and *E,E*-α-farnesene in the essential oil from the above ground parts in *C. maculatum* [7]. The synthesis of furocoumarins was induced in leaves and seedlings stressed with the heavy metal Cu [8]. Callus cultures obtained from leaf stalks produced furocoumarins when they are treated with various elicitors as alginic acid, cellulase, chitosan, ethylene, methyl jasmonate, salicylic acid, copper (II) sulphate and silver nitrate [9]. These compounds act as protectants against phytopathogens.

The plant produces thick tap roots, however, only little is known about the phytochemistry of these underground plant parts. A report from Serbia presents steroids from *C. maculatum* roots [10]. Therefore, the aim of the present work was to study which characteristic compounds typical for Apiaceae occur in *C. maculatum* roots, and to assess their variability in plants of different origin.

## 2. Results

The bioactive compounds of *C. maculatum* have been analyzed in roots dichloromethane extracts by GC-MS. The samples came from single plants of 16 accessions grown on an experimental field. Ten compounds could be evaluated. There were five furocoumarins xanthotoxin (8-methoxy- psoralen), isopimpinellin (5,8-dimethoxy-psoralen), bergapten (5-methoxy-psoralen), psoralen and marmesin, two prenylated coumarins as osthol and *trans*-suberenol, the aliphatic C_17_-polyines (polyacetylenes) falcarinol ((Z)-Heptadeca-1,9-diene-4,6-diin-3-ol) and falcarindiol ((Z)-Heptadeca-1,9-diene-4,6-diin-3,8-diol) and, finally, the phenylpropanoid elemicin. The mass spectra of falcarinol (55 (100), 91 (66), 43 (64), 115 (43), 117, (35), 77 (30), 129 (27), 131 (26), 78 (23), 193 (23), 81 (22), 29 (22)) and falcarindiol (55 (100), 129 (72), 41 (66), 91 (62), 43 (58), 128 (54), 77 (54), 115 (44), 79 (32), 105 (25), 157 (25), 57 (22)) were in good accordance with the published literature [11,12,13]. Additionally, the furanocoumarin columbiatenin and a suberenol isomer were only found in few samples and therefore not further considered.

Table 1 presents an overview over all 152 analyzed samples and shows that over all samples a broad variability as manifested by high standard deviations occurred for all compounds. Falcarindiol was recorded in highest concentrations, followed by xanthotoxin and isopimpinellin. For marmesin, suberenol and elemicin usually low contents were measured. All compound showed a positive skewness, indicating that there were more low values than high values. The positive kurtosis values indicate a higher and narrower curve than normal distribution [14].

A more detailed view of the repartition of the compounds for the individual accessions is given in Table 2. Additionally, within the accessions, a great variability was present for all compounds, visible in high standard deviations. Accessions A9, A5 and A10 had the highest falcarindiol means, A7, A14 and A16 being the lowest. Accessions A9, A4, A3, A18, A5 and A4 were rather high in xanthotoxin, the remaining low. *Trans*-suberenol was absent in accessions A2, A3, A4, A8, A9, A11 and A12, while elemicin could not be found in A8 and A12.

**Figure 1:**
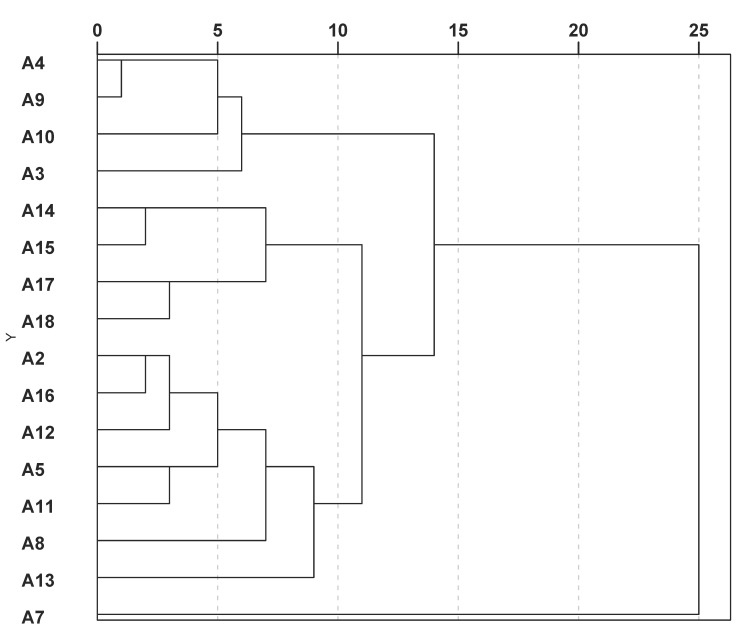
Dendrogram showing similarities between accessions of *Conium* roots according to the mean values of 10 analyzed compounds.

**Figure 2:**
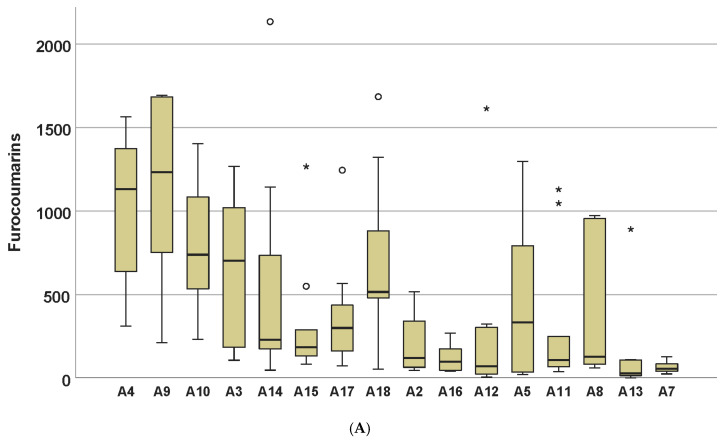

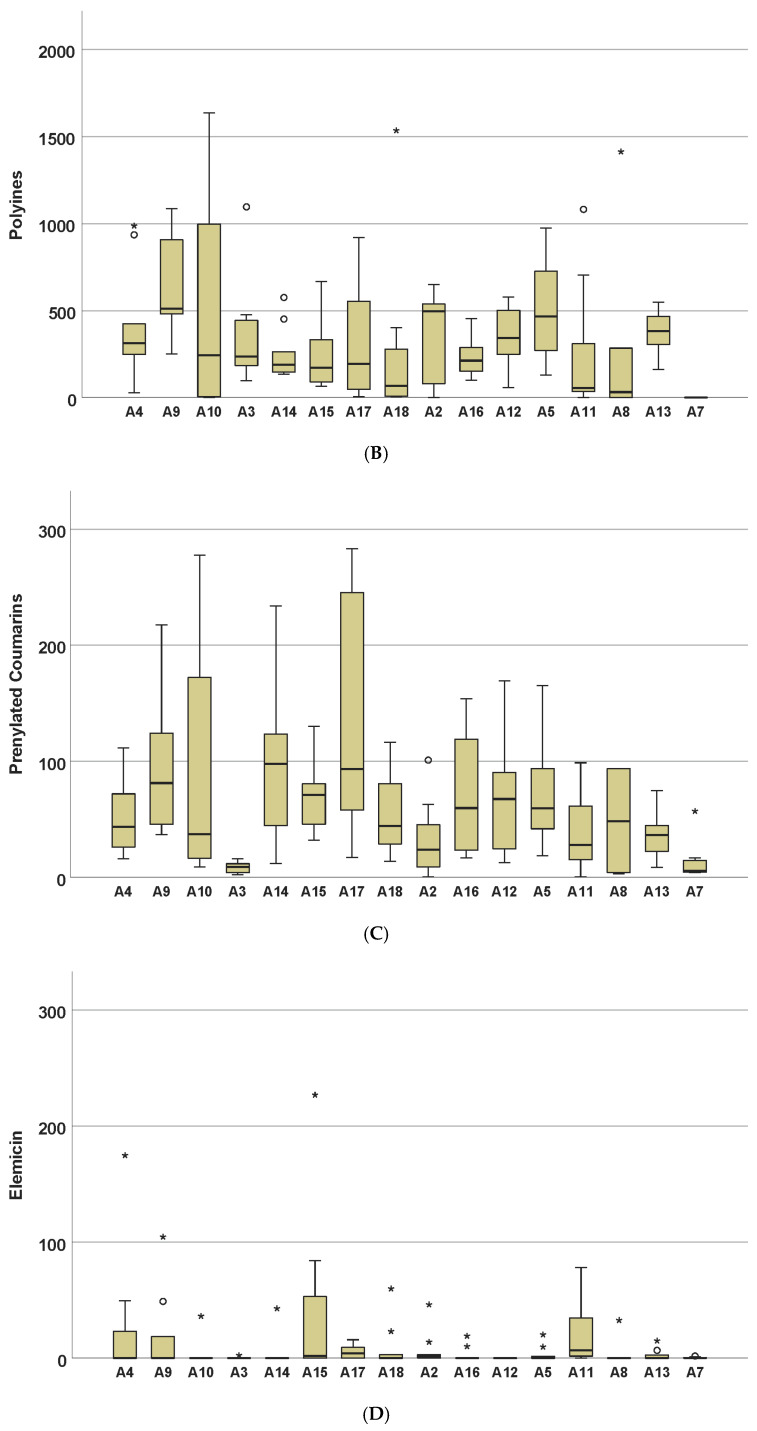
Boxplots presenting the variability of compound classed in 16 accessions (µg/g). * and o mark outliers. Order of accessions according to hierarchical cluster analysis (HCA) analysis in Figure 1. (**A**): furocoumarins; (**B**): polyines; (**C**): prenylated coumarins; (**D**): elemicin.

In order to obtain a possible classification of the accessions, a hierarchical cluster analysis (HCA) was performed. Figure 1 represents the corresponding dendrogram, and the boxplots of Figure 2 gives the variability of the compound classes for the 16 accessions.

The dendrogram suggests a classification in four groups. The first group on the upper part comprising accessions A3, A4, A9 and A10 had higher furocoumarin contents. The second group gathering A14, A15, A17 and A18 had medium contents of furocoumarins and higher contents of prenylated coumarins, especially *trans*-suberenol. The third group with A2, A5, A8, A11, A12, A13 and A16 appeared rather low in furocoumarins. The last group at the bottom is represented by accession A7 alone. This accession was generally low in all compounds. No correlation could be found with geographic origins of the samples.

**Figure 3:**
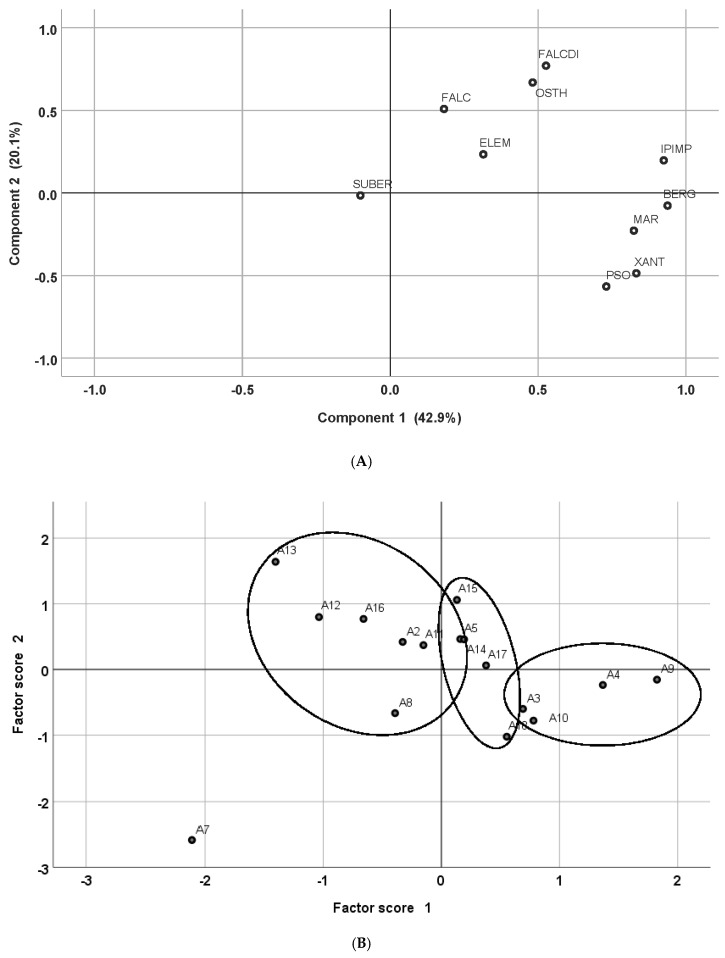
Loading plots of the compounds (**A**) and scoring plots of the accession means (**B**) of the first two components in principal component analysis (PCA). BERG: bergapten, ELE: elemicin, FALC: falcarinol, FALCDI: falcarindiol, IPIMP: isopimpinellin, MAR: marmesin, OST: osthol, PSO: psoralen, SUBER: suberenol, XANT: xanthotoxin.

Computing a principal component analysis (PCA) gave an additional view on the complex variability between and within the accessions. In this analysis, the 10 compounds as variables have been reduced to four components, with eigenvalues greater than one accounting for 86.4% of the variability. The first two components accounted for 64.4% of the variability. Corresponding biplots are presented in Figure 3.

On the loading plot of the first two components, all compounds but suberenol had a positive component 1 loading, and the highest loadings were recorded for the furocoumarins. Falcarindiol had a high component 2 loading. The compounds falcarinol, *trans*-suberenol and elemicin, which occurred rather in low concentrations in the sample, had lower loadings on both components. On the scoring plot comparing the accessions, a grouping partially similar to those suggested by HCA could be found. Accession A7 having lowest contents of active compounds was isolated on the lower left quadrant. The third group in HCA (A2, A5, A8, A11, A12, A13 and A16) with a high ratio of falcarindiol to furocoumarins appeared mainly in PCA on the upper left quadrant, while the remaining accessions had all positive factor 1 values. In this plotting, the highest factor 1 value was assigned to A9, which was the accession with the highest total active compound content (Table 2).

**Figure 4:**
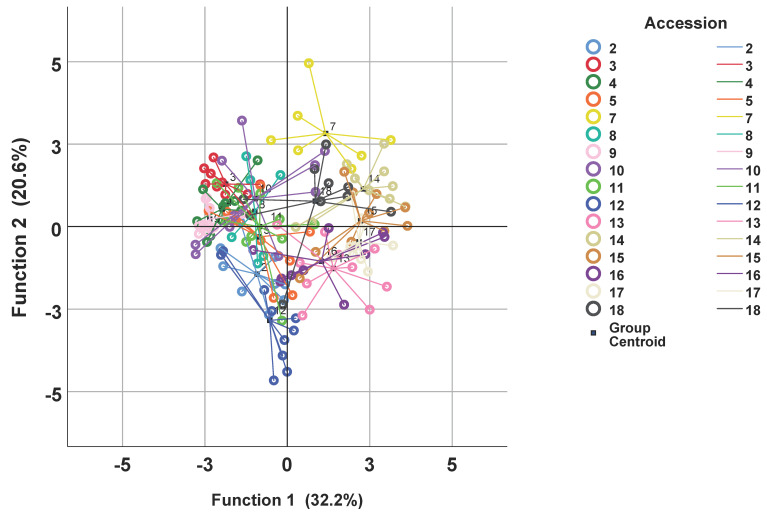
Discriminant scores of the two first functions grouping the samples to the accessions.

Finally, to visualize the relation between individual samples of the accessions a canonical discriminant analysis (CDA) was performed. In this multivariate analysis, samples had to be assigned to the accessions as predefined groups. Figure 4 plots the values of the first two discriminant functions for the samples and the corresponding accessions. Some accessions were well separated as, for instance, A3, A12 and A17, but there were also many overlaps. This can be attributed to the high variability within the accessions. Altogether, 63.8% of the samples were correctly classified to their accessions.

## 3. Discussion

A discussion of bioactive compounds in *Conium maculatum* should at first concern the well documented toxic alkaloids. However, in alkaline lipophilic extracts of various root samples, we failed to prove the presence of alkaloids. In root tips from seedlings histochemical tests showed the presence of alkaloids [16]. However, another study also pointed out that *Conium* roots were devoid of alkaloids [4].

All compounds showed high variability between and within the accessions. High variability of secondary compounds within a plant species seems not to be uncommon. For instance, in fresh celeriac and parsnip root collected from the Czech market total furocoumarin content varied from 1 to 50 mg/kg and 1 to 140 mg/kg, respectively [17]. Within a wild growing population of *Silaum silaus* myristicin, the main volatile in the fruits from single plants, ranged from 131 to 12550 μg/g, a nearly 100-fold difference between lowest and highest contents [18].

The highest levels of falcarindiol were recorded in the roots, while falcarinol appeared relatively low. For *C. maculatum*, small amounts of falcarinol (0.4%) were reported in the inflorescence essential oil in plants from Serbia [6]. Falcarinol and related polyacetylenes are widely distributed in the Apiaceae family [19]. Commercially available vegetables as *Apium graveolens*, *Daucus carota* and *Pastinaca sativa* contained on a dry weight basis 230 to 1620 mg/kg falcarinol and 240 to 5770 mg/kg falcarindiol [20]. In carrots, falcarinol and falcarindiol contributed to the bitter taste off which occurred in cold stored carrots and carrot puree [12]. Falcarinol ranged from 8.1 to 27.5 mg/kg and falcarindiol from 21.7 to 84.3 mg/kg in fresh carrots, and was dependent from the carrot cultivars [13]. Another report mentioned on dry weight basis 315 mg/kg falcarindiol and 82.2 mg/kg falcarinol in the carrot variety Blanche [11]. Furthermore, parsley roots contained 403 mg/kg falcarindiol and 629 mg/kg falcarinol, and *Pastinaca sativa* 240 and 165 mg/kg of these two compounds, respectively, all on dry weight basis [11]. So, the present *C. maculatum* roots appear to contain comparable amounts of falcarindiol and less falcarinol than common Apiaceae root vegetables. Aliphatic C_17_-polyacetylenes of the falcarinol type display a variety of interesting biological activities, including antibacterial, antimycobacterial and antifungal effects as well as anti-inflammatory and anti-platelet-aggregatory properties [19]. Additionally, neuritogenic and neuroprotective effects of falcarinol have been reported [19]. Falcarindiol exerts a potent modulatory action on GABA_A_-receptors [21]. Finally, anticancer activity has been demonstrated for falcarindiol and falcarinol. These compounds were able to reduce the number of neoplastic lesions, as well as the growth rate of the polyps in rat gut, suggesting a preventive effect on the development of colorectal cancer [22].

The Apiaceae species represent a major plant family able to produce furocoumarins. For a long time, it has been known that these compounds, once applied onto the skin and exposed to light, induce burns and lesions of the skin. Psoralene-type linear furocoumarins, like xanthotoxin and bergapten, show strong such photosensitizing effects, in contrast to angular furocoumarins. This toxicity is dependent on their ability to form DNA adducts under the influence of UV-A, leading to cross-links in DNA and, ultimately, resulting in a potent cytotoxicity and acute inflammation [23,24]. The treatments of psoriasis and vitiligo are pharmaceutical applications of furocoumarins. Further noticeable effects of furocoumarins have been observed. Xanthotoxin proved acetylcholinesterase inhibitory activity [25]. In the mouse maximal electroshock-induced seizure test, xanthotoxin had clear-cut anticonvulsant activity [26]. Antiproliferative activities on cancer cell lines have been demonstrated for a flower extract from the umbelliferous *Magydaris tomentosa*, which was rich in xanthotoxin, xanthotoxol, isopimpinellin and bergapten [27].

In an ecological context, furocoumarins are defense chemicals against insect herbivores and microbial attacks. The synthesis of the compounds might be induced by the attack. The ability of *C. maculatum* to produce furocoumarins has been demonstrated by the induction of their synthesis through various elicitors in callus cultures. Isopimpinellin, bergapten, xanthotoxin and psoralen were the main elicited compounds [9]. The present study deals with these same four compounds. Additionally, the callus cultures produced low amounts of oroselone, coloumbiatenin and marmesin [9]. Actually, low amounts of marmesin were found in low levels in most of the accessions, while columbiatenin occurred only in very few root samples (data not shown). Recently, a study reported anticancer activity of marmesin in human leukemia cells [28].

To evaluate the levels of furocoumarins in the present *C. maculatum* roots a comparison with other Apiaceae species can be attempted. These compounds occur in a range of umbelliferous vegetables and herbs. In fresh celeriac and parsnip roots from the Czech Republic, the average furocoumarin content was 17 and 26 mg/kg, respectively [17]. Calculated on a dry weight basis, these values can be considered as 4–6 times higher. Carrots usually have low levels of furocoumarins as for instance 0.068 mg/kg fresh weight [29], or less than 0.05 mg/kg [30].

Leaves of various parsley varieties had, in their leaves, 1.6–9.6 mg/kg xanthotoxin, 1.9–52.7 mg/kg psoralene and 19.9–479 mg/kg bergapten in the dry matter [31]. The outer, older celery leaves (*Apium graveolens*) contained up to 44.9 mg/kg fresh weight linear furocoumarins, levels that were high enough to threaten human health. The roots of these plants having 0.9 mg/kg of these compounds were considered as safe [32]. During storage of parsnip roots the levels of furocoumarins usually increase in dependence of the storage conditions [17,33]. Taken together, the present *C. maculatum* roots contained comparable levels of furocoumarins as parsnip, but higher levels than carrots.

For prenylated coumarins also some remarkable activities have been reported. Suberosin and suberenol isolated from *Ferulago carduchorum* showed anticoagulant properties, but no acute or subchronic toxicity when administered to rats as they prolonged the prothrombin time [34]. Research on osthol demonstrated various effects as anti-asthmatic, antidiabetes, antiseizure and improving mental disorders. Therefore osthol might be useful in treating epilepsy [35].

The phenylpropanoid elemicin as alkenyl-benzene has a terminal double bound on the side chain. Compounds having this structure display a genotoxic and carcinogenic potency. Elemicin occurs also in plants like nutmeg or parsley, which are used as flavorings and spices. Risk assessment for food products containing such plants have been attempted [36,37].

In sum, *C. maculatum* roots contain a complex bouquet of active compounds in comparable amounts as in other Apiaceae species. Interactions between individual compounds are to be expected and need further research.

## 4. Materials and Methods

### 4.1. Plant Material

Sixteen accessions of poison hemlock were grown in field plots at the Leibniz Institute of Plant Genetics and Crop Plant Research (IPK) in Gatersleben, Germany (Table 3). The plots were established in 2014, and the harvest of the roots took place in August 2015, at maturity of the plants when the fruits of the terminal umbels were fully developed. From each accession, 10 root samples were taken from single plants. From accessions 2, 7 and 8 only 9, 7 and 6, plants could be harvested so that altogether 152 samples were analyzed. The roots were kept at −80 °C until further analysis.

### 4.2. Extraction of the Active Compounds

Pieces 2–3 g of the roots from single plants were dried overnight at 35 °C and ground to a fine powder using a laboratory mill. Then a portion of 0.5–1 g was extracted with 6 mL dichloromethane for 120 min in an ultrasonic bath. The solvent dichloromethane contained hexadecane at a rate of 0.05 mg/mL as internal standard. After centrifugation, 3 mL of the resulting extract were gently reduced to dryness and finally taken up in 300 µL dichloromethane.

### 4.3. Gas Chromatography/Mass Spectrometry (GC/MS)

The analyses were carried out on an Agilent Technologies 7890A gas chromatograph equipped with a 5975 C quadrupole mass selective detector, (Agilent Technologies, Santa Clara, CA, USA). The separation was done on a 60 m × 0.25 mm fused silica column coated with 0.25 μm HP5-MS. Helium was the carrier gas at a velocity of 1.2 mL/min in the constant flow mode. One µL was injected in the injection port heated at 270 °C and a split ratio of 15:1. The oven temperature was programmed at a rate of 12 °C/min from 60 °C to 210 °C, and then at a rate of 5 °C/min from 210 °C to 280 °C. The final temperature hold time at 280 °C lasted 12 min. The transfer line to the mass selective detector was heated at 280 °C and the scan range was *m*/*z* 40–450, with 1.86 scans/s. Mass spectra were used for the identification of the compounds, by comparison with the entries of spectra libraries NIST08 and Wiley 275 and the further literature [11,12,13,39]. Additionally, retention indices were calculated in comparison to the n-alkanes C_8_–C_30_ and compared with the literature data [39]. Quantitative calculations were based on the response of the internal standard hexadecane and an estimation of response factors for the individual compounds (Table S1).

For the quantitative evaluation, the total ion current (TIC) was used, and it was assumed that all compounds gave the same response as the internal standard hexadecane. By this method, based on the signal to noise ratio, in the dried root, limit of detection (LOD) and limit of quantification (LOQ) were between 0.3 and 0.7 mg/kg and 1.0 and 2.7 mg/kg, respectively (Table S1). Inter and intraday repeatability varied between 15–20% for the various compounds.

### 4.4. Statistical Analysis

The statistical analyses were done with the package IBM SPSS for Windows, version 26.0 (IBM Corporation, Armonk, NY, USA). In the data matrix, values below LOD were assumed as 1/3 LOD and values between LOD and LOQ as 1/3 LOQ. Many samples had low contents and high variance of the various compounds occurred so that skewed distributions were observed. Therefore, the values were transformed according to: y = Log(x), with y transformed and x original value. Means and standard deviations for the individual accessions were first calculated with the logarithmic values and then back-transformed [15].

The complex interplay of the compounds was further analyzed by multivariate methods. A hierarchical cluster analysis (HCA), using the squared Euclidian distance with linkage between groups, was calculated to visualize similarities between the accessions where the logarithmic population means were taken as cases. A principal component analysis (PCA) was calculated similarly. Finally, to visualize the variability within the populations, a plotting of a canonical discriminant analysis (CDA) was performed.

## 5. Conclusions

Ten major active compounds could be evaluated in the roots of a range of *C. maculatum* accessions. A remarkably high variability between and within the accessions could be observed, which could be the basis for studying biological activity of the compounds. This stresses also the need for a comprehensive characterization of a plant material when it is used to study biological effects. A further aspect might be investigating a possible differentiated response to environmental conditions [40].

The compounds were typical for Apiaceae, including furocoumarins, prenylated coumarins, aliphatic C_17_-polyacetylenes and phenylpropanoids, and showed a wide variability. The calculated concentrations were in the same order of magnitude as reported for other Apiaceae roots, including edible vegetables. Nevertheless, the compounds may display pronounced bioactivity, as the literature suggests. However, as the roots did not contain alkaloids, no or only a low toxicity can be assumed for these plant parts.

## Figures and Tables

**Table 1:** Selected statistical characteristics for 152 samples from 16 accessions.

Accession	XANT	IPIM	BER	PSO	MAR	OST	SUBE	FALC	FALD	ELE
Mean	254.4	168.9	79.1	68.1	12.5	52.7	11.0	9.0	328.8	9.6
Median	76.9	89.3	48.3	9.5	0.0	36.6	0.0	1.4	253.1	0.0
SD	391.6	197.3	84.1	141.1	25.6	58.1	23.0	26.2	315.4	28.1
Min.	0.0	0.0	0.0	0.0	0.0	0.0	0.0	0.0	0.0	0.0
Max.	2297.4	940.7	450.1	979.8	145.5	407.6	111.4	248.8	1570.2	227.0
Skewness	2.8	1.8	2.0	3.7	3.2	2.6	2.7	7.0	1.5	5.1
Kurtosis	10.3	3.0	4.3	17.3	11.4	9.7	7.0	56.9	2.5	31.7

Mean, median, min and max in mg/kg dry matter; Min. 0.0: lower than limit of detection; XANT: xanthotoxin, IPIM: isopimpinellin, BER: bergapten, PSO: psoralen, MAR: marmesin, OST: osthol, SUBE: *trans*-suberenol, FALC: falcarinol, FALD: falcarindiol, ELE: elemicin.

**Table 2:** Active compounds in 16 *Conium maculatum* accessions (mg/kg).

Accession		XANT	IPIM	BER	PSO	MAR	OST	SUBE	FALC	FALD	ELE
A2	Median	12.9	65.2	45.2	0.0	0.0	23.8	0.0	1.9	483.8	1.7
N = 9	Mean *	16.1	72.6	43.5	0.9	0.8	14.7	0.0	0.7	155.0	1.2
	SD *	4.1	2.4	2.1	10.0	9.6	6.3		7.6	7.7	8.9
A3	Median	418.5	177.0	101.4	114.3	9.2	8.8	0.0	18.9	215.5	0.0
N = 10	Mean *	218.6	115.2	72.9	83.8	4.5	7.3	0.0	17.5	246.5	0.2
	SD *	3.5	2.3	2.4	4.4	8.8	2.0		3.5	1.9	2.4
A4	Median	474.4	363.3	100.4	72.0	30.7	43.3	0.0	3.3	308.4	0.0
N = 10	Mean *	484.3	317.4	82.0	77.9	8.6	40.2	0.0	0.9	299.8	1.3
	SD *	2.2	2.0	3.2	2.6	13.5	1.9		10.6	2.7	18.3
A5	Median	201.7	91.1	50.1	54.7	0.0	59.4	0.0	1.0	465.6	0.0
N = 10	Mean *	89.4	64.3	36.7	11.5	0.6	55.8	0.3	0.7	443.7	0.6
	SD *	8.4	4.0	3.0	19.5	8.8	2.0	3.2	4.8	1.9	6.8
A7	Median	39.8	3.3	11.6	6.8	0.0	5.6	4.5	0.0	0.0	0.0
N = 7	Mean *	34.4	2.7	7.6	9.7	0.0	2.3	2.9	0.0	0.2	0.3
	SD *	2.5	3.8	5.4	3.8		5.6	7.46		4.6	3.1
A8	Median	37.6	67.0	37.9	9.7	0.0	48.4	0.0	0.0	32.0	0.0
N = 6	Mean *	34.0	79.1	42.0	8.2	0,4	27.7	0.0	0.2	5.5	0.4
	SD *	2.9	7.3	2.5	3.1	4.0	7.1		9.8	87.7	8.9
A9	Median	767.8	252.8	171.5	215.5	23.2	81.2	0.0	1.4	509.6	0.0
N = 10	Mean *	643.7	244.0	144.0	229.2	20.1	81.9	0.0	0.9	552.1	1.3
	SD *	2.2	2.1	2.1	2.4	6.2	1.9		7.6	1.6	16.8
A10	Median	386.3	160.3	144.0	75.1	0.0	36.9	0.0	0.0	243.3	0.0
N = 10	Mean *	420.9	162.7	147.2	46.5	1.0	37.1	0.7	0.3	52.9	0.3
	SD *	2.1	3.0	1.9	8.2	7.5	4.1	7.0	9.6	27.4	5.6
A11	Median	56.0	27.3	37.2	6.1	0.0	27.5	0.0	7.2	50.3	6.8
N = 10	Mean *	47.6	33.9	41.0	3.3	0.7	19.9	0.2	2.6	69.0	5.7
	SD *	6.5	4.0	2.3	13.4	9.6	6.0	1.5	10.3	7.4	9.5
A12	Median	0.6	49.8	22.4	0.0	0.0	67.4	0.0	0.0	342.6	0.0
N = 10	Mean *	2.8	24.6	23.2	0.7	0.3	49.2	0.0	0.1	299.7	0.0
	SD *	20.6	11.5	3.9	10.7	3.9	2.3		2.9	2.0	
A13	Median	1.6	20.3	6.1	0.0	0.0	31.8	1.3	7.2	347.4	0.0
N = 10	Mean *	2.0	18.8	8.5	0.3	0.0	28.2	1.0	10.5	335.4	0.5
	SD *	10.5	5.4	5.6	1.7		1.9	5.5	3.8	1.4	6.4
A14	Median	98.0	110.6	52.2	16.5	0.0	31.7	54.3	8.2	185.1	0.0
N = 10	Mean *	96.2	121.0	60.5	11.8	0.4	33.3	21.4	9.7	206.1	0.3
	SD *	4.5	2.5	3.0	9.9	5.2	1.9	8.9	2.7	1.6	5.9
A15	Median	42.1	110.0	33.4	0.5	0.0	44.9	26.1	13.8	158.6	1.9
N = 10	Mean *	45.8	117.5	45.6	1.7	0.7	47.9	11.5	5.5	166.5	2.3
	SD *	3.3	2.1	2.0	11.5	4.9	1.6	4.5	9.1	2.1	18.1
A16	Median	18.9	49.3	33.8	0.0	0.0	52.2	3.6	1.2	211.7	0.0
N = 10	Mean *	15.7	50.9	21.2	0.4	0.7	33.5	1.5	0.7	205.7	0.4
	SD *	3.0	2.0	1.6	4.1	6.9	4.8	8.1	9.1	1.6	6.8
A17	Median	54.5	139.8	60.9	8.6	11.4	61.3	20.6	0.0	195.0	4.2
N = 10	Mean *	55.2	143.0	62.8	5.2	3.0	35.0	31.3	0.2	140.2	1.6
	SD *	2.3	2.5	2.3	5.8	10.1	8.5	2.1	5.5	4.9	7.8
A18	Median	313.7	190.7	47.9	11.2	28.8	16.5	22.2	0.0	67.1	0.0
N = 10	Mean *	241.5	137.5	57.9	9.6	16.8	11.8	7.5	0.3	57.2	0.6
	SD *	4.0	2.3	2.2	8.9	6.6	5.1	13.1	8.9	8.1	10.6

* Calculated from logarithmic values and then back transformed according to [15]. XANT: xanthotoxin, IPIM: isopimpinellin, BER: bergapten, PSO: psoralen, MAR: marmesin, OST: osthol, SUBE: *trans*-suberenol, FALC: falcarinol, FALD: falcarindiol, ELE: elemicin. 0.0: below limit of detection.

**Table 3:** List of *Conium maculatum* accessions from the Leibniz Institute of Plant Genetics and Crop Plant Research (IPK).

Nr	Accession	Species	Date	Country of Origin	Acquisition from
A2	CONI 2	*Conium maculatum*	1952	unknown	BG Bucharest
A3	CONI 3	*Conium maculatum*	1953	unknown	BG Krakow
A4	CONI 4	*Conium maculatum*	1975	France	BG Frankfurt
A5	CONI 5	*Conium maculatum*	1990	Georgia	Georg. SSR
A7	CONI 7	*Conium maculatum*	2002	Germany	BAZ
A8	CONI 8	*Conium maculatum*	2002	Germany	BAZ
A9	CONI 9	*Conium maculatum*	2002	Germany	BAZ
A10	CONI 10	*Conium maculatum*	2000	Russia	BG Halle/Saale
A11	CONI 11	*Conium maculatum*	2002	Germany	BAZ
A12	CONI 12	*Conium maculatum*	2002	Germany	BAZ
A13	CONI 13	*Conium maculatum*	2002	Germany	BAZ
A14	CONI 14	*Conium maculatum*	2002	Germany	BAZ
A15	CONI 15	*Conium maculatum*	2002	Germany	BAZ
A16	CONI 16	*Conium maculatum*	2002	Germany	BAZ
A17	CONI 17	*Conium maculatum*	2002	Italy	BAZ
A18	CONI 18	*Conium maculatum*	2002	Germany	BAZ

For further details see [38]. Date refers to year of acquisition of the respective accession. (BG: Botanical Garden; BAZ: Braunschweig Genetic Resources Centre).

## Supplementary Materials

The following are available online at https://www.mdpi.com/2223-7747/9/8/939/s1, Table S1: Characteristics of secondary metabolite analysis.
