# Supplementary material for: Diversity of Secondary Metabolites in Roots from Conium maculatum L
Source: Plants (Basel). 2020 Jul 24;9(8):939. doi: 10.3390/plants9080939 (PMC7464025; doi:10.3390/plants9080939)
Supplement: Supplementary file 1 [file plants-09-00939-s001.pdf]

Supplementary Materials for

Diversity of secondary metabolites in roots from *Conium maculatum* L.

Remigius Chizzola, Ulrike Lohwasser

Table S1. Characteristics of secondary metabolite analysis.

Compound	Formula	MW	Ion	RI	RF	LOD	LOQ
Xanthotoxin	$C_{12}H_8O_4$	216	216	2082	1.81	0.7	2.7
Isopimpinellin	$C_{13}H_{10}O_5$	246	246	2280	1.85	0.7	2.8
Bergapten	$C_{12}H_8O_4$	216	216	2106	1.73	0.7	2.6
Psoralen	$C_{11}H_6O_3$	186	186	1877	1.64	0.7	2.5
Marmesin	$C_{14}H_{14}O_4$	246	246	2356	1.59	0.6	2.4
Osthol	$C_{15}H_{16}O_3$	244	244	2168	1.42	0.6	2.1
Suberenol	$C_{15}H_{16}O_4$	260	189	2300	1.54	0.6	2.3
Falcarinol	$C_{17}H_{24}O$	244	55	2039	0.70	0.3	1.0
Falcarindiol	$C_{17}H_{24}O_2$	260	129	2199	0.76	0.3	1.1
Elemicin	$C_{12}H_{16}O_3$	208	208	1559	1.15	0.5	1.7

MW: Molecular weight (g/mol); Ion: specific ion used to refine LOD; RI: Retention index on a HP5-MS column relative to the n-alkanes; RF: Response factor relative to the internal standard Hexadecane; LOD and LOQ: Limit of detection and limit of quantification based on signal/noise ratios 1 to 3 and 1 to 10, respectively, and specific ion traces.
